# hnRNP E1 and E2 have distinct roles in modulating HIV-1 gene expression

**DOI:** 10.1186/1742-4690-4-28

**Published:** 2007-04-23

**Authors:** Kathryn Woolaway, Kengo Asai, Andrew Emili, Alan Cochrane

**Affiliations:** 1 Department of Molecular and Medical Genetics, University of Toronto Toronto, Ontario M5S 1A8, Canada

## Abstract

Pre-mRNA processing, including 5' end capping, splicing, and 3' end cleavage/polyadenylation, are events coordinated by transcription that can influence the subsequent export and translation of mRNAs. Coordination of RNA processing is crucial in retroviruses such as HIV-1, where inefficient splicing and the export of intron-containing RNAs are required for expression of the full complement of viral proteins. RNA processing can be affected by both viral and cellular proteins, and in this study we demonstrate that a member of the hnRNP E family of proteins can modulate HIV-1 RNA metabolism and expression. We show that hnRNP E1/E2 are able to interact with the ESS3a element of the bipartite ESS in tat/rev exon 3 of HIV-1 and that modulation of hnRNP E1 expression alters HIV-1 structural protein synthesis. Overexpression of hnRNP E1 leads to a reduction in Rev, achieved in part through a decrease in *rev *mRNA levels. However, the reduction in Rev levels cannot fully account for the effect of hnRNP E1, suggesting that hmRNP E1 might also act to suppress viral RNA translation. Deletion mutagenesis determined that the C-terminal end of hnRNP E1 was required for the reduction in Rev expression and that replacing this portion of hnRNP E1 with that of hnRNP E2, despite the high degree of conservation, could not rescue the loss of function.

## Introduction

Prior to their export to the cytoplasm, eukaryotic pre-mRNAs undergo a number of processing events that include capping, splicing and 3' end processing (cleavage of the nascent transcript and polyadenylation). These processing events occur as the transcript is being synthesized, and each one is able to influence the efficiency and specificity of the others [1-5]. RNA processing is also required for efficient export of the mRNA to the cytoplasm and its translation [6-10]. Retroviruses such as HIV-1 however, export unspliced and incompletely spliced viral RNAs normally retained in the nucleus. By encoding proteins from these intron containing RNAs, retroviruses increase their coding potential [11]. Indeed, suboptimal splicing of the primary HIV transcript generates over 30 mRNAs, many of which (e.g. Gag, Gagpol, Vpr, Vif, Vpu and Env) contain introns. HIV is able to overcome the requirement for complete splicing of a transcript prior to its export to the cytoplasm by the action of the virally encoded protein Rev. Rev functions by forming multimers that interact directly with a *cis*-acting Rev response element (RRE). This complex is exported via an interaction with host cellular Crm1/Exportin 1 through a pathway normally used by snRNA [12,13].

Numerous host cellular factors can influence the processing and transport of HIV-1 viral RNAs, including Sam68 [14-17], hStaufen [18,19], eIF5A [20,21] and hRIP [22]. hnRNPs are another class of proteins that associate with nascent transcripts to influence many stages of RNA metabolism [23-25], including that of HIV-1 [26-31]. The hnRNP E proteins (or α-complex proteins (αCPs) or poly(C)-binding proteins (PCBPs)) were first functionally characterized as components of a complex that stabilizes human α-globin [32-34]. Subsequent studies demonstrated the existence of five major isoforms, hnRNP E1 to hnRNP E4 and hnRNP E2-KL (a splice variant of hnRNP E2 differing from the original by a 31 amino acid deletion in the region between KH2 and KH3), encoded by four genetic loci [35,36]. Since their initial characterization, hnRNP E proteins have been implicated in a wide array of processes including mRNA stabilization, translational enhancement and translational silencing [32-34]. Studies have also indicated a role for the hnRNP E proteins in the post-transcriptional regulation of a number of viruses. Examples include the stabilization and translational enhancement of poliovirus RNA, and translational silencing of human papillomavirus L2 mRNA [37-39].

The hnRNP E proteins belong to the triple KH domain containing protein family that includes hnRNP K [32]. All of the isoforms of hnRNP E contain three repeats of the type 1, 70 amino acid KH domain, designated KH1, KH2 and KH3. Each KH domain is able to interact independently with a target RNA sequence. Therefore, these proteins have the potential for complex and highly specific RNA interactions [40]. The hnRNP E proteins are expressed in many human tissues and have been shown to bind poly C regions [35,41,42], although both hnRNP E1 and hnRNP E2 have been shown to interact with mRNAs of low C content [38]. The most highly expressed and well characterized of the hnRNP E proteins are the hnRNP E1 and E2 isoforms, which are 89% similar at the amino acid level [40]. While multiple splice isoforms of hnRNP E2 are known to exist, hnRNP E1 is encoded by an intronless gene believed to be the product of a retrotransposition event of a fully processed minor isoform of hnRNP E2 [42]. It has been suggested that the conservation of hnRNP E1 indicates it serves a non-redundant function although, it is presently unclear what this role might be.

In this study, we began to look for endogenous *trans*-acting factors that acted on HIV-1 ESS3. Two of the factors identified, were hnRNP E1 and hnRNP E2 and in this paper, we investigate the effects of overexpression and depletion of hnRNP E1 and E2 on HIV-1 gene expression. We demonstrate that overexpression of hnRNP E1 but not hnRNP E2 can inhibit expression of the Rev-dependent RNAs encoding gp120 and p24. Our data is consistent with hnRNP E1 acting to decrease viral mRNA translation. Depletion of either hnRNP E1 or E2 resulted in increased production of HIV-1 structural proteins. Domain analysis of the protein indicates that only the two carboxy-terminal KH domains of hnRNP E1 are required for its inhibitory effect. The differential capacity of hnRNP E1 and E2 to modulate HIV-1 RNA processing and utilization suggest that, despite their high degree of similarity, these two proteins have distinct, non-redundant roles in modulating HIV-1 gene expression.

## Results

### hnRNP E proteins are enriched in ESS3a containing columns

**Figure 1:**
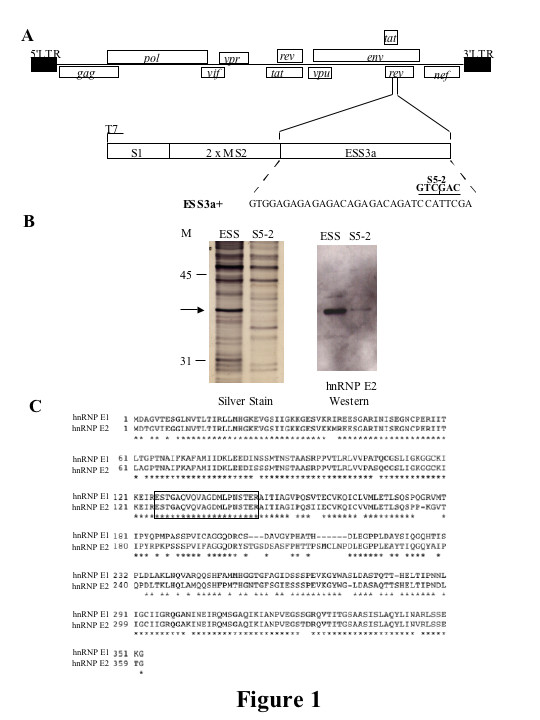
**Identification of *trans*-acting factors that associate with ESS3a**. RNA affinity columns were programmed withTRAP-tagged RNA transcripts of ESS3a or with a mutant of ESS3adesignated ESS3a 5-2. HeLa nuclear extract was passed over the columns and column eluates fractionated by SDS-PAGE and silver stained. ESS3a enriched bands were excised and sequenced by mass spectrometry. (A) Schematic representation of HIV-1 genome. Open boxes represent the open reading frames encoding the indicated viral proteins, shaded boxes indicate 5' and 3' LTRs. Also shown is a schematic of the TRAP-ESS vector. The construct contains a Streptavidin binding aptamer, S1 and two MS2 coat protein binding sites followed by the ESS3a sequence. The sequence of ESS3a is shown. The altered nucleotides of the mutant designated ESS3a 5-2 are indicated above the wild-type sequence. (B) Silver stain of proteins eluted from Streptavidin column with indicated RNA bait. Proteins were fractionated on 10% SDS-PAGE. The arrow indicates the excised band. Western blot against hnRNP E2; ESS or mutant thereof are as shown (C) Comparison of hnRNP E1 and hnRNP E2. Boxed is the sequence generated from the excised band analyzed by mass spectrometry.

Previous work by this lab has shown hnRNP A1 to be one component of a complex that modulates the activity of the bipartite exon splicing silencer (ESS3) found in tat/rev exon 3 [26]. However, mutagenesis studies suggest that binding of hnRNP A1 to the ESS3 alone is not sufficient for full activity, indicating that other factors may play a role in mediating ESS3 activity [26]. In a continuation of this work, we attempted to identify additional factors that associate with ESS3. RNA affinity columns were programmed with tandem RNA affinity purification (TRAP)-tagged RNA transcripts of ESS3a or a mutant of ESS3a, designated ESS3a 5-2, that results in partial loss of ESS3 function without affecting hnRNP A1 binding [26]. The TRAP tag consists of a Streptavidin binding aptamer, S1 and two MS2 coat protein binding sites (Figure 1A). HeLa nuclear extract was passed over the columns and eluates fractionated by SDS-PAGE, then silver stained (Figure 1B). Proteins enriched in the wt ESS3a sample were excised and sequenced by mass spectrometry.

One of the sequences generated by mass spectrometry matched a region found in both hnRNP E1 and hnRNP E2 (Figure 1C). We obtained an antibody to hnRNP E2 (kind gift from Raul Andino) and western blotting analysis confirmed that hnRNP E2 was selectively retained on the wt ESS3a column (Figure 1B), at this stage we did not have an antibody to hnRNP E1. Work by several groups has demonstrated a role for members of the hnRNP E family in the post-transcriptional regulation of a number of viruses [37-39]. Therefore, further study of the effects of this family of *trans*-acting factors on HIV expression appeared justified.

### hnRNP E1 But Not hnRNP E2 overexpression suppresses HIV-1 gene expression

Vectors expressing myc epitope tagged forms of hnRNP E1 and hnRNP E2 (mycE1, mycE2) were generated and confirmed by sequencing. These were co-transfected into 293 cells alongside the replication incompetent proviral HxBru R-/RI- construct and CMVPLAP plasmid expressing secreted alkaline phosphatase (SEAP). 48 hrs post transfection, cells were harvested and lysates analyzed by western blot. To compare the level of expression of mycE1 and mycE2, myc blots were performed. Equal loading was confirmed by blotting for tubulin. To determine whether any effects on virus expression were as a result of a general inhibition of translation, expression of secreted alkaline phosphatase (SEAP) was also measured.

**Figure 2:**
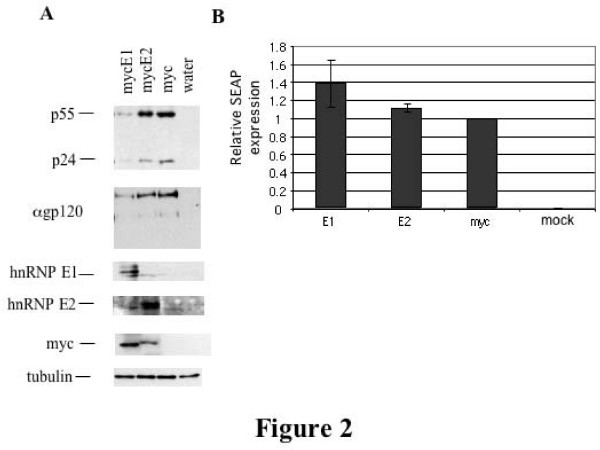
**hnRNP E1 but not E2 overexpression decreases HIV-1 structural protein levels**. 293 cells were transfected with mycE1, mycE2 or the empty CMVmyc vector, alongside provirus HxBruR-/RI- andCMVPLAP. 48 hrs post transfection, cells were lifted in 1×PBS and a fraction (1/4) of the cells lysed in 9 M urea, 5 mM Tris pH8 and fractionated on SDS-PAGE gels. Protein was transferred to PVDF membrane and probed with antibody to p24. Blots were stripped in 62.5 mM TrisHCl pH 6.7, 2%SDS, 100 mM β-mercaptoethanol and reprobed with antibodies to gp160/120 (α gp120), hnRNP E1 and E2, myc and tubulin as indicated. (A) Results of western blotting. Antibodies and transfected plasmids are as indicated. (B) Relative SEAP expression in transfected cells. Levels were normalized to that of the empty CMVmyc vector control. Results shown are an average of three experiments. Error bars represent standard deviation.

As shown in Figure 2, overexpression of mycE1 results in the inhibition of expression of the HIV-1 proteins p55, p24 and gp160/120, while overexpression of mycE2 has little to no effect. The peptide expressed by the control vector (CMVmyc 3xterm) is too small for detection by western blotting. Some of the difference in response could be attributed to the lower expression of mycE2 relative to myc E1. However, higher expression of mycE2 failed to alter HIV expression (data not shown). Modulation of HIV protein expression by mycE1 appears to be restricted, as no significant effect on expression of co-transfected SEAP was observed (Figure 2B). Thus, hnRNP E1 and E2 differ in their ability to inhibit HIV-1 gene expression.

### hnRNP E1/E2 overexpression has limited effects on HIV-1 RNA levels and splicing

**Figure 3:**
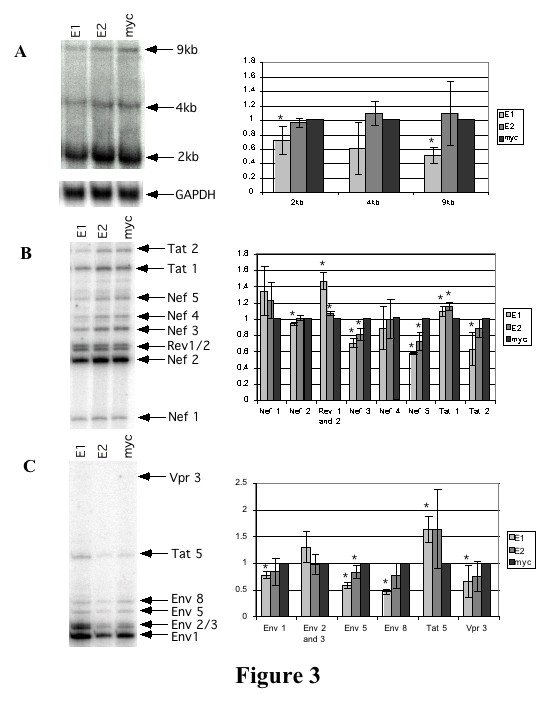
**hnRNP E1 and E2 overexpression have only limited effects on HIV-1 RNA levels**. 293 cells were transfected with eithermycE1 (E1), mycE2 (E2) or CMVmyc (myc) along with provirus HxBruR-/RI- and CMV PLAP. 48 hrs post transfection, cells were lifted in 1×PBS and total RNA isolated. Northern and RT-PCRs were performed. (A) Northern analysis of the effect of mycE1/E2 overexpression on levels of 2 kb, 4 kb and 9 kb classes of HIV-1 RNA. Probe derived from the HIV-1 LTR was used to detect all 3 classes of viral RNA; viral RNAs (9, 4, 2 kb) are indicated. Blots were stripped and reprobed for GAPDH RNA to confirm equal loading. At right, a summary of 3 sets of experimental results quantitated using Image Quant v5.2. Figures are normalized to GAPDH with the results for the myc control vector set at 1 for each RNA class. Error bars show standard deviation. An asterisk denotes 95% confidence level that the difference between the sample and the myc control is significant. (B) RT-PCR showing effects of mycE1 and mycE2 on the 2 kb species of HIV-1 RNA. Reporter plasmids and identity of amplicons are indicated. At right, a summary of 3 sets of experimental results quantitated using Image Quant v5.2. Shown is the percentage of each species of the total RNA for each lane. For each species of RNA the myc value was set at 1. Error bars show standard deviation. An asterisk denotes 95% confidencelevel that the difference between the sample and the myc control is significant. (C) RT-PCR showing effects of mycE1 and mycE2 on the 4 kb species of HIV-1 RNA. Reporter plasmids and identity of amplicons are indicated. At right, a summary of 3 sets of experimental results, quantitated using Image Quant v5.2 as described in (B).

In light of the interaction of hnRNP E1/E2 with ESS3 and the impact of hnRNP E1 on HIV-1 protein expression, we anticipated that the factor might function through effects on viral RNA processing. Therefore, we examined the effect of mycE1/E2 overexpression on virus RNA abundance and processing. 293 cells were transfected with HIV-1 provirus and mycE1, mycE2, or myc control vectors, total RNA isolated and analyzed by Northern blot. Blots were hybridized with probe corresponding to the LTR region found in all 3 classes (2, 4 and 9 kb) of HIV-1 RNA. Equal loading was confirmed by reprobing for GAPDH mRNA. Analysis of multiple trials revealed that overexpression of mycE1 results in a small decrease in all 3 classes of HIV-1 RNA (Figure 3A). In contrast, overexpression of mycE2 had no effect on HIV-1 RNA levels. The effect of overexpressing mycE1 on the splicing pattern of the 2 kb and 4 kb classes of HIV-1 RNA was determined by RT-PCR (see [43] for more information on HIV splicing). Overexpression of mycE1 or mycE2 had only minor effects on HIV-1 splice site selection (Figures 3B, C). None of the changes in HIV-1 RNA levels observed could readily account for the significant reduction in viral protein expression induced by hnRNP E1 overexpression.

### hnRNP E1 or E2 knockdown increase HIV-1 gene expression

**Figure 4:**
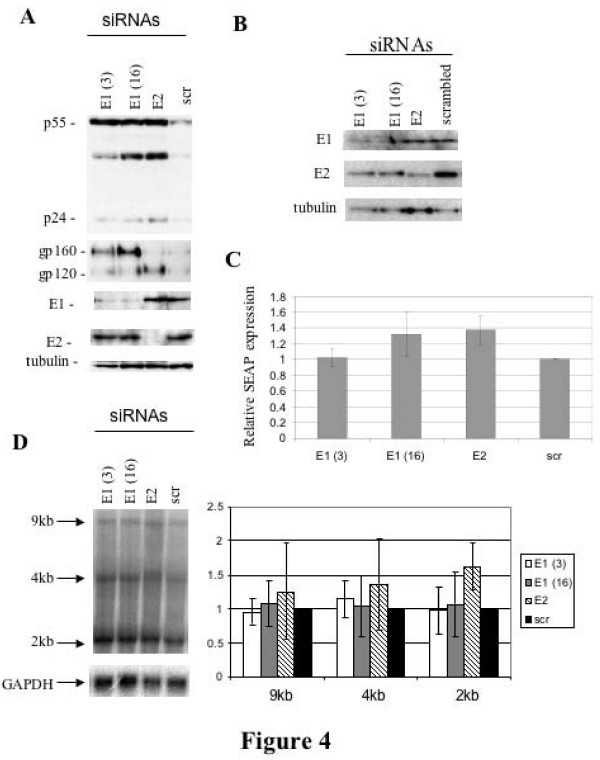
**Depletion of hnRNP E1 and E2 alters HIV-1 gene expression**. 293 cells were transfected with siRNAs to hnRNP E1(E1(3) or E1(16)), hnRNP E2 (E2) or a scrambled siRNA control (scr)using Oligofectamine. 24 hrs later, cells were transfected withHxBruR-/RI- provirus and CMVPLAP using Fugene. 48 hrs post transfection, cells were harvested, lysates fractionated on SDS-PAGE gels and analyzed by western blot. (A) Results of western blotting. Proteins examined and siRNAs used are as indicated. (B) Kinetics of siRNA depletion of hnRNP E1/E2. Cells were treated with control siRNA (scrambled) or directed to hnRNP E1 (E1(3), E1(16)), or hnRNP E2 (E2) and harvested 24 h after transfection. Cell extracts were fractionated on SDS PAGE gels, transferred to PVDF membrane and probed with antibody to hnRNP E1 (E1), hnRNP E2 (E2), or tubulin (tubulin). (C) Relative SEAP expression in transfected cells. Media was harvested 48 hrs post transfection and assayed for SEAP activity. Levels were normalized to that of the scrambled siRNA control. Results shown are an average of three experiments. Error bars represent standard deviation. (D) Northern analysis of the effect of hnRNP E1/E2 depletion on HIV RNA levels. Cells were treated with siRNAs as above and total RNA extracted. Following fractionation on formaldehyde agarose gels and transfer to nitrocellulose membrane, blots were probed to detect viral RNAs (9, 4, and 2 kb). Blots were reprobed for GAPDH RNA to confirm equal loading. Quantitation of 3 experiments is shown at right. Results were normalized to GAPDH with the data for the scrambled siRNA sample (scr) set at 1. Error bars show standard deviation.

To complement the overexpression assays, the effect of depleting endogenous hnRNP E1/E2 by siRNA was also assessed. As shown in Figure 4B, depletion of each protein was achieved within 1 day of the addition of the siRNAs. Depletion of hnRNP E1 or E2 resulted in shifts in the expression of HIV-1 structural proteins (Fig. 4A). Depletion of hnRNP E1 or E2 resulted in increased synthesis of HIV-1 Gag (p55, p24) and Env (gp160/120). Analysis of co-transfected SEAP (Figure 4C) revealed a modest increase in expression upon deletion of either hnRNP E1 or hnRNP E2, suggesting that loss of either protein has a general small, but stimulatory effect on protein synthesis (maximum 1.4 fold). This finding could partly explain the effect seen with HIV-1 protein expression. Subsequent analysis by Northern blots on viral RNA abundance failed to reveal any significant alterations on viral RNA abundance that would account for the effects observed (Fig. 4D). Analysis of individual splicing events by RT-PCR also failed to reveal any changes upon depletion of either hnRNP E1 or E2 (data not shown).

### hnRNP E1 overexpression reduces rev synthesis

**Figure 5:**
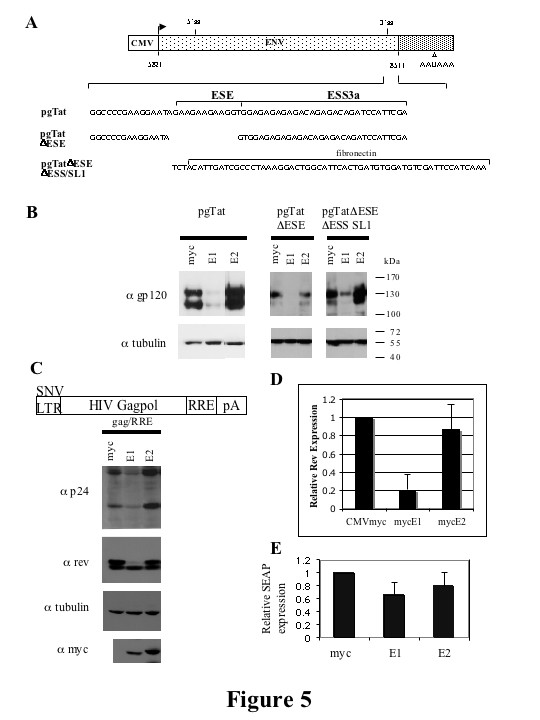
**hnRNP E1 but not hnRNP E2 inhibit expression of subgenomic HIV-1 expression vectors**. (A) Schematic of HIV-1 *env *expression vector, pgTat, including the location of the ESE and ESS. The sequences of pgTat derivatives pgTatΔESE and pgTatΔESEΔESS SL1 are shown. (B) HeLa cells were co-transfected with pgTat or derivatives thereof, SVH6Rev, and hnRNP E expressing plasmids as indicated. 48 hours post-transfection, cells were harvested in RIPA buffer, and lysates fractionated on SDS-PAGE gels. Following transfer to PVDF membranes, blots were probed with antibody against gp120 and tubulin. (C) Effect of hnRNP E proteins on Gag/RRE expression. Shown is a diagram of the Gag expression construct used. HeLa cells were transfected with Gag/RRE, hnRNP E and Rev expression constructs as indicated. 48 hrs post transfection, cells were harvested, and lysates fractionated on SDS-PAGE gels. Blots were probed with antibody against Rev and p24, stripped then reprobed for tubulin and myc to confirm equal loading and similar expression of myc-tagged constructs respectively. To quantitate changes in Rev protein expression, blots were scanned and analyzed in Imagequant. Summary of 5 independent determinations is shown (D). (E) Effect of hnRNP E proteins on SEAP expression. Media from cells transfected with a SEAP expressing plasmid was harvested 48 hrs post transfection and SEAP expression normalized to that seen upon cotransfection with the CMVmyc control vector. Results shown are an average of 3 experiments. Error bars show standard deviation. SEAP expression of the myc control vector was set to 1.

The failure of myc E1/E2 overexpression and depletion of endogenous hnRNP E1/E2 to significantly alter HIV RNA processing consistent with modulation of ESS3 function lead us to examine the response in greater detail. In particular, we wanted to find the basis for hnRNP E1's drastic inhibition of viral protein synthesis. Given that both ESE3 and ES3 encode portions of Rev and Env within the HIV-1 provirus, testing the role of these sequences in mediating the response to hnRNP E1 is problematic. To overcome this obstacle, mycE1 and mycE2 were co-transfected into HeLa cells along with pgTat and the Rev expressing plasmid SVH6Rev to determine if similar results could be obtained using this HIV reporter construct [44]. pgTat is an env-expressing reporter that contains the first coding exon of Rev, but lacks much of the second coding exon (see [26] for more information). Therefore, export of the intron containing *env *RNA and subsequent env expression requires *trans *expression of Rev. As shown in Figure 5B, co-transfection of mycE1 with pgTat and Rev resulted in a decrease in the level of gp160/120 expression, while co-transfection with mycE2 had little or no effect, consistent with the results from provirus transfections.

Two pgTat derivatives, pgTatΔESE (lacking the ESE but retaining the ESS) and pgTatΔESEΔESS SL1 (where the ESE and ESS have been replaced with a sequence of similar size from the EDA exon of the human fibronectin gene; Figure 5A) were also used to assess the potential role of ESE and ESS3a in mediating any effects of mycE1/E2 on HIV RNA processing [26]. In all cases, mycE1 overexpression reduced gp160/120 expression while mycE2 overexpression had no effect. Consistent with previous results [45], a low level of gp160/120 expression was observed from pgTatΔESE which was further reduced upon expression of mycE1. The results demonstrate that the inhibitory effect of mycE1 does not require either ESE3 or the ESS3a, as reduced gp160/120 expression was observed for all constructs tested (Figure 5B).

To probe the basis for the observed inhibition of gp160/120 expression, we first looked at whether hnRNP E1 was altering viral RNA processing, in particular preventing the formation of unspliced/cleaved *env *RNA, the preferred substrate for Rev export [17,46]. However, only a two-fold decrease in the level of unspliced, polyadenylated env RNA was seen upon overexpression of mycE1 (data not shown). This observation is consistent with the reduction in proviral RNA seen in Figure 3A, but it is unable to fully account for the loss of viral protein expression. Alternatively, mycE1 may act indirectly by affecting Rev, as export of *env *RNA is dependent upon Rev. Indeed, co-expression of mycE1 decreased the number of Rev positive cells as assayed by immunofluorescence (data not shown). To test this further, we transfected cells with the Rev-dependent reporter, GagRRE, and assessed expression of both Gag (α p24) and Rev by Western blot in the presence or absence of the myc-tagged hnRNP E proteins. As shown in Figures 5C and 5D, cotransfection of mycE1 with the GagRRE reporter construct reduced both p24 production and Rev expression. In contrast, mycE2 had no effect. No significant change in cotransfected SEAP expression was seen, suggesting that the inhibition of Rev expression by mycE1 is not a generalized effect (Figure 5E). Thus, hnRNP E1 and E2 isoforms appear to differ in their ability to inhibit HIV-1 gene expression both from the provirus and from HIV-1 expression constructs. Also, the response to mycE1 appears to be independent of ESS3 within the HIV-1 env reporter. However, the sequence of ESS3 is also present in Rev, possibly explaining why hnRNP E1 regulates its expression.

### Deletion of the C terminal KH domain of hnRNP E1 abrogates its ability to inhibit Rev expression

**Figure 6:**
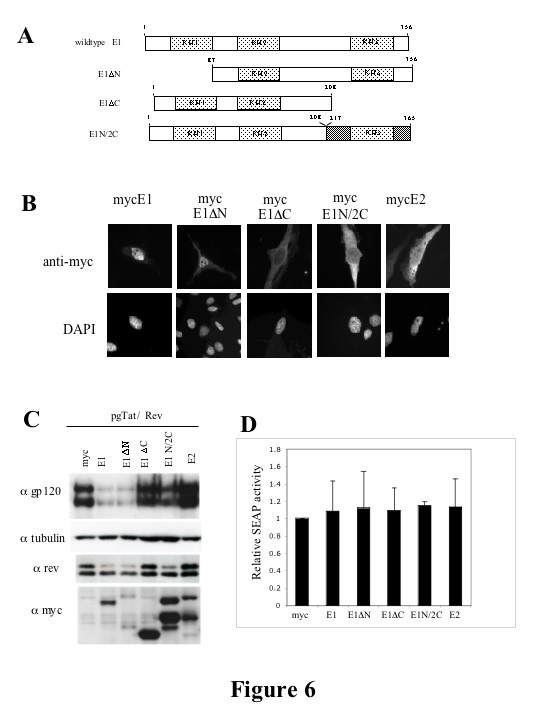
**Suppression of HIV-1 gene expression by hnRNP E1 is dependent upon the C-terminal KH domain**. (A) Schematic of hnRNP E1 and domain mutants thereof. Light grey boxes denote the KH domains. Dark shading denotes hnRNP E2 sequence and white indicates hnRNP E1 sequence. (B) Localization of hnRNP E1 and domain mutants and hnRNP E2. HeLa cells were grown on coverslips and transfected with the hnRNP E expressing plasmids. 48 hrs post transfection, cells were washed with 1× PBS, fixed in 4% paraformaldehyde, 1× PBS and localization of transfected hnRNP E proteins (anti-myc) and nuclei (DAPI) determined. Magnification is 630×. Shown are representative examples of results obtained in multiple trials. (C) 293T cells were transfected with mycE1/E2 expressing plasmids as indicated along with pgTat and SVH6Rev. 48hrs post transfection, cells were harvested and lysates fractionated on SDS-PAGE gels. Following transfer to PVDF membranes, blots were probed with antibody to gp120, tubulin, rev or the myc tag. Cell supernatants were analyzed for levels of SEAP expression (D).

The marked difference in ability of mycE1 versus mycE2 to modulate HIV-1 expression despite their high degree of similarity (89% at the amino acid level, see Fig. 1C) led us to map the domains involved. A series of deletion mutants were constructed (Figure 6A). Deletion mutants of mycE1 which lack the N-, or C-terminal KH domains were designated mycE1ΔN and mycE1ΔC, respectively. A domain swap mutant was also generated consisting of the C-terminal KH domain of hnRNP E2 with the N-terminal KH domains 1 and 2 of hnRNP E1 (mycE1N/2C). Expression of the mutants was confirmed by Western blot (see 6C, α myc). Localization of the domain mutants was determined by immunofluorescence (Figure 6B). Full length mycE1 is predominately localized to the nucleus (Figure 6B), and deletion of the N-terminal KH domain (amino acids 1–86) did not alter this localization pattern. However, it was noted that mycE1ΔN localized to discrete foci within the nucleus generating a speckled appearance. In contrast, deletion of the C-terminal 148 amino acids (mycE1ΔC) resulted in a pronounced redistribution of the protein to the cytoplasm. Replacement of the C-terminal domain of hnRNP E1 with that of hnRNP E2 (mycE1N/2C) failed to restore the nuclear accumulation of the protein, the distribution being similar to that of mycE2 (localized diffusely throughout the cell (Figure 6B)).

Given the effect of the N- and C-terminal domain mutants on hnRNP E1 subcellular distribution, we were interested to determine if these changes in protein localization correlated with a change in the ability of mycE1 to suppress viral protein expression. 293T cells were transfected with the env expression vector (pgTat), as well as plasmids expressing Rev and mycE1 (or domain mutants thereof) or mycE2. As shown in Figure 6C, mycE1ΔN mutant retains the ability to inhibit gp160/120 and Rev expression (Figure 6C). In contrast, deletion of the C-terminal KH domain of hnRNP E1 completely abrogates the ability of the protein to inhibit gp160/120 and Rev expression. Interestingly, the domain swap mutant mycE1N/2C had reduced activity relative to mycE1 indicating that the C-terminus of hnRNP E1 performs a unique function. As before, no effect on Rev or gp160/120 expression was observed upon co-expression with mycE2. None of the constructs induced any significant alteration in expression of cotransfected secreted alkaline phosphatase (SEAP, Fig. 6D). Thus, the C-terminal KH domain of hnRNP E1 is required both for the localization of the protein to the nucleus and, in part, for the inhibition of viral protein (gp160/120, Rev) expression.

### Effects of hnRNP E1 and its domain mutants on HIV-1 RNA abundance, processing and subcellular distribution

**Figure 7:**
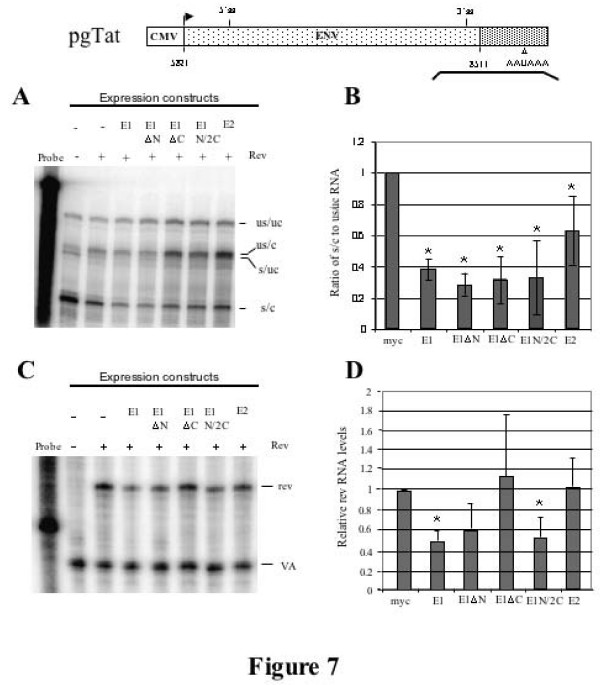
**hnRNP E1 overrexpression selectively affects spliced HIV-1 *env *RNA levels**. (A) Shown is a diagram of the pgTat vector with the line below indicating the position of the probe (spanning both the 3'ss and polyadenylation sequence) used for RPA analysis. 293T cells were transfected with hnRNP E expressing plasmids as indicated, alongside pgTat and SVH6Rev. RNA was isolated 48 hours post transfection and used in RNase protection assays. Protection products were resolved on denaturing PAGE gels. Analysis of 3' end processing of unspliced *env *RNA by RNase protection assay. Identities of the various bands observed are indicated; unspliced, uncleaved (US/UC); unspliced, cleaved (US/C); spliced, uncleaved(S/UC); spliced, cleaved (S/C). (B) Graphical representation of the effect of hnRNP E proteins on the ratio of s/c to us/uc RNA. Averages of a minimum of three independent experiments are shown. Asterisk denotes values that were deemed significantly different from control at p < 0.05 (C) 293T cells were transfected with hnRNP E expressing plasmids as indicated, alongside Gag/RRE and SVH6Rev. 48 hrs post transfection RNA was isolated and used in RNase protection assays. Analysis of *rev *RNA levels by RNase protection assay. *rev *RNA abundance was corrected using the cotransfected VA control (pSPVA) and normalized to the vector control indicated by (-). (D) Summary of the effect of N- and C-terminal KH domain mutants of hnRNP E on *rev *RNA levels. Averages of a minimum of three independent experiments are shown. Asterisk denotes values that were deemed significantly different from control at p < 0.05

We next looked at the effect of overexpressing hnRNP E1 or its domain mutants on splicing and cleavage/polyadenylation in the context of the subgenomic reporters. To do this, a probe that spans both the 3' ss and the polyadenylation signal of pgTat was used (see Fig. 7). Four protection products are generated corresponding to: unspliced/uncleaved (US/UC), unspliced 3' end processed RNA (US/C), spliced/uncleaved (S/UC), and fully processed spliced/cleaved RNA (S/C). Protections generated were of the expected size by comparison to molecular weight markers [17] and experiments with RNA from mock transfected cells failed to generate protection products (data not shown). Examination of *env *RNA in the presence of mycE1 and mycE1ΔN revealed a slight decrease in the level of unspliced/cleaved RNA (US/C; Figure 7A). However, the greatest effect seen was a reduction in the levels of spliced, cleaved (S/C) in the presence of mycE1, indicating that this factor acts in a manner similar to that seen with the provirus (Figures 3A and 7B). All constructs tested yielded some reduction in spliced/cleaved (S/C) RNA levels, the least active being mycE2. Little change in unspliced/uncleaved (US/UC) *env *RNA is apparent in the presence of mycE1 indicating that the effect of the protein is limited to a subset of the viral RNAs generated.

To investigate the basis for the effect of mycE1 on Rev expression, its effect on *rev *RNA abundance was also examined. Total RNA was isolated from 293T cells transfected with pgTat, and plasmids expressing Rev, and mycE1, mutants thereof, or mycE2, and RPAs performed using a *rev *specific probe. Co-transfection of a VA RNA expression vector served as an internal control. mycE1, mycE1ΔN, and mycE1N/2C resulted in only a modest (~2 fold) decrease in the level of *rev *RNA (Figure 7C, D). No significant changes in *rev *RNA levels were detected upon cotransfection with mycE1ΔC, or mycE2. Reduction in RNA levels is observed even for those constructs lacking an intron (SV Rev), consistent with an alteration in stability of the affected RNAs.

**Figure 8:**
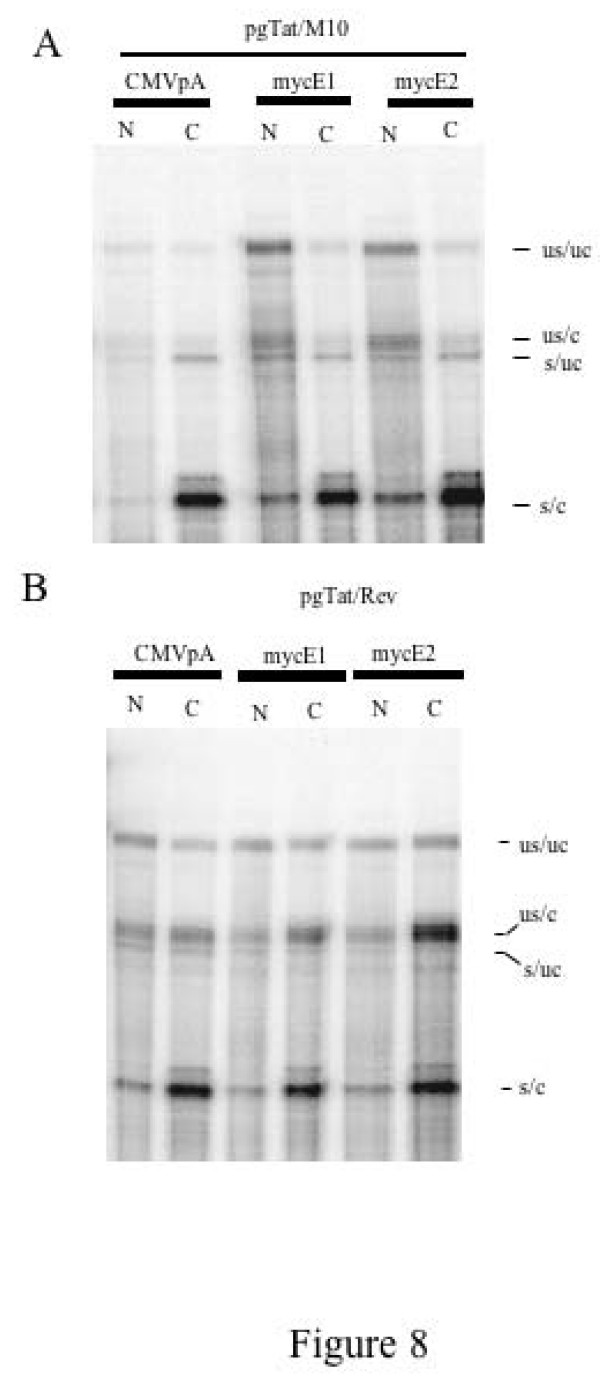
**Effect of hnRNP E1/E2 Overexpression on the Subcellular Distribution of Viral RNAs**. 293T cells were transfected with control (CMVpA) or mycE1/E2 expression plasmids as indicated, alongside pgTat and either an inactive form of Rev (M10) (A) or wild type Rev (B). Following harvest, nuclear and cytoplasmic fractions were prepared, RNA extracted and used in RNase protection assays. Protection products were resolved on denaturing PAGE gels. Identities of the various bands observed are indicated; unspliced, uncleaved (US/UC); unspliced, cleaved (US/C); spliced, uncleaved (S/UC); spliced, cleaved (S/C).

Given that reduction in Rev levels might be expected to affect the cytoplasmic accumulation of the viral RNAs encoding the structural proteins, we examined the effect of mycE1 and mycE2 expression on the subcellular distribution of pgTat RNAs. As shown in Fig. 8A, cotransfection of pgTat with an inactive form of Rev (M10) [47] resulted in little accumulation of the US/C form of pgTat in the cytoplasm. In contrast, co-transfection of wild type Rev induced accumulation of US/C form of pgTat RNA in the cytoplasm. In addition, despite the reduction in Rev expression, co-expression of mycE1 or mycE2 failed to prevent the transport of the viral RNA to the cytoplasm (Fig. 8B). Therefore, sufficient levels of Rev are present in the presence of mycE1 to export viral RNAs. However, translation of the viral RNA in the cytoplasm must be prevented to explain the marked reduction in gp160/120 expressed observed in the presence of mycE1.

## Discussion

In this study, we have demonstrated that hnRNP E1 is a *trans*-acting factor able to modulate HIV-1 gene expression. This fact, coupled with the identification of hnRNP E1 in the virion particle, suggests that this factor plays an important role in HIV-1 expression and assembly [48]. Although hnRNP E1 were initially examined due to an interaction with ESS3 *in vitro*, subsequent work determined that it does not function through altering the splicing of HIV-1 RNA to any significant extent (Fig. 3B, C). Indeed, hnRNP E1 overexpression led to dramatic suppression of reporter constructs (GagRRE, pgTatΔESEΔESS/SL1) that lacked ESS3 (Fig. 5). However, since all viral RNAs include the terminal exon containing ESS3, the effect of hnRNP E1 can be explained by its interaction with the mRNAs encoding the HIV-1 regulatory proteins Tat and Rev. Although the ESS3 lacks a polycytosine tract similar to the consensus hnRNP E1 binding site, other studies have demonstrated binding to sequences that do not fit the consensus [38]. In addition, recent work has shown that hnRNP E1 can be recruited to an RNA through protein-protein interaction (in particular via hnRNP A2) to modulate translation of a mRNA [49]. Given the previously determined role of hnRNP A1/A2 in mediating the activity of ESS3 [50,51], recruitment of hnRNP E1/E2 to this sequence may be indirect. However, the loss of hnRNP E1/E2 binding to mutants of ESS3 that retain hnRNP A1 binding (Fig. 1) and the independence of the response to the presence of the ESS3 suggests that hnRNP E1 may be interacting with sequences elsewhere in the viral RNA. Previous work on hnRNP E1 and E2 has identified roles for these factors in the translational control of both viral and cellular mRNAs [33,37-39,52-54] as well as in the mediation of mRNA stabilization [55-57]. These effects are the result of either steric protection of the mRNA 3'UTR from endonucleolytic attack [58] or protection of the poly (A) tail from degradation by interacting with the poly(A)-binding protein (PABP; [59-61]). Little is known as to whether the different hnRNP E proteins have unique functions or if they show redundancy in their actions. In this study, we establish that hnRNP E1 and hnRNP E2 elicit different responses as only overexpression of hnRNP E1 inhibited the expression of several HIV-1 genes. This difference was observed in the context of both the HIV provirus and subviral expression plasmids. The failure to detect significant alterations in cotransfected SEAP expression suggests that the response to hnRNP E1 is somewhat restricted. Consistent with the overexpression assays, depletion of hnRNP E1 or E2 by siRNA resulted in increased expression of HIV-1 Gag and Env. These findings underline the differential roles of these factors in modulating HIV-1 gene expression.

The basis for the observed effect of hnRNP E1 remains only partially defined. Overexpression of hnRNP E1 resulted in a decrease in the levels of Rev protein. The loss of Rev is explained in part by effects at the RNA level. Forms of hnRNP E1 (mycE1 and mycE1ΔN) that yielded reduced Rev protein expression elicited a 2 fold reduction in *rev *RNA abundance. A similar change in *rev *RNA but not protein levels was also observed upon co-expression of mycE1N/2C. However, the fold change in *rev *RNA levels is not consistent with the extent of reduction in Rev protein (decreased 5 fold, Fig, 5D). Therefore, it is likely that the loss of Rev expression is also due to reduced translation of its RNA, a phenomenon consistent with the known role of hnRNP E1 in translational silencing of RNAs in other systems. A similar decrease in abundance is also seen for the spliced form (S/C) of pgTat (Fig. 7) and proviral RNAs (Fig. 3) although, in the context of pgTat, all constructs except hnRNP E2 reduced S/C RNA abundance by ~2 fold. However, despite reduced Rev expression, viral RNAs encoding structural proteins still accumulate in the cytoplasm (Fig. 8). Therefore, the loss of HIV Gag and Env expression upon hnRNP E1 overexpression is not due to a block in export of these RNAs but rather an inhibition of their translation.

Deletion analysis identified domains of hnRNP E1 essential for the modulation of HIV-1 expression and revealed a correlation with the extent of nuclear accumulation of the protein. Analysis of the subcellular localization of our hnRNP E1 domain mutants is consistent with earlier studies [40] that identified a nuclear localization signal (NLS) between KH2 and KH3 of hnRNP E1. Deletion of the C-terminal 148 amino acids from hnRNP E1 (mycE1ΔC) resulted in the redistribution of the protein from the nucleus to the cytoplasm. Removal of the C-terminal KH domain also resulted in loss of inhibition of HIV-1 gene expression. However, our work failed to show localization of full length hnRNP E1 to nuclear speckles. Swapping the C-terminal domain (KH3) of hnRNP E1 and E2 (mycE1N/2C) only partially restored the nuclear accumulation of the mutant, the distribution of the fusion protein being comparable to that observed for mycE2. The failure of mycE1N/2C to inhibit Rev and p24 expression suggests that the C-terminal domains of hnRNP E1 and E2 are not functionally interchangeable and implicate that sequence differences in this region partially account for the different activities of these two proteins.

In summary, despite high levels of sequence identity between the hnRNP E1 and E2 isoforms, the emerging model is that these proteins are capable of fulfilling distinct cellular functions. These differences are reflected in the failure of domain swaps to maintain the activity of the proteins. The isoform specific effects for these highly similar proteins is surprising. Work by other groups has also suggested differential roles for hnRNP E1 and hnRNP E2. This includes the finding that hnRNP E1 has a greater affinity for AUF/hnRNP D than hnRNP E2 [61], as well as differential regulation of hnRNP E1 and E2 in response to hypoxic stressing of cortical neurons [62]. This observation supports the notion that despite a high degree of similarity at the amino acid level, these proteins possess non-redundant functions. To our knowledge, the present work is among the few examples of a system that allows for examination of the unique effects of the hnRNP E isoforms. Further analysis using this system may allow us to elucidate the amino acid differences between hnRNP E1 and E2 that account for their differential effect on HIV-1 RNA metabolism and refine the mechanism by which hnRNP E1 modulates HIV-1 expression.

## Materials and methods

### Expression constructs

The following plasmids have been previously described: SVH6Rev, pgTat, pgTatΔESE, pgTatΔESEΔESS SL1, pgTatΔESE S5-2, Gag RRE, pSPVA, HxBruR-/RI- [26,47]. pTRAP plasmid was generously provided by Dr. H. Krause, University of Toronto. CMVmycE1 was constructed by amplifying HeLa cDNA with primers hnRNP E1-F-Hind (5' CCC AAG CTT ATG GAT GCC GGT GTG ACT GAA 3') and hnRNPE1-R-Pst (5' AAA ACT GCA GCT AGC TGC ACC CCA TGC CCT T 3'). The resulting amplicon was digested with HindIII/PstI and cloned into the corresponding sites of the CMVmyc3xterm vector (a N-terminal myc-tagged CMV immediate early promoter, with a 3xterm cassette and SV40 polyadenylation signal in a Bluescript (Stratagene) backbone). CMVmycE2 was constructed by amplifying HeLa cDNA with primers hnRNPE2-F-Hind (5' CCC AAG CTT ATG GAC ACC GGT GTG ATT GAA 3') and hnRNPE2-R-Bam (5' CGC GGA TCC CTA GCT GCT CCC CAT GCC ACC 3'). The resulting amplicon was HindIII/BamHI digested and cloned into the corresponding sites of the CMVmyc3Xterm vector. CMVmycE1ΔN was constructed by amplifying CMVmycE1 with primers E1Δ1FHind (5' CCC AAG CTT ATG ACC AAC AGT ACC GCG GCC 3') and hnRNPE1-R-Pst. Amplicons were HindIII/PstI digested and cloned into the corresponding sites of CMVmyc3xterm. CMVmycE1ΔC was constructed by digesting CMVmycE1 with XbaI and religating the backbone. CMVmycE1N/2C was constructed by ligating the 500 bp fragment generated upon XbaI digest of CMVmycE2 with the CMVmycE1 XbaI digested backbone. pTRAP ESS and S5-2 were constructed by PCR amplification of pgTatΔESE and pgTatΔESE S5-2 with primers ESS-F (5' CCC AAG CTT GGG ATC CCC GAA GAA ATA GTG G 3') and ESS-R (5' CCG CTC GAG GCC AAG GTC TGA AGG TCA CTC GA 3'). Amplicons were digested with HindIII and XhoI and cloned into the corresponding sites of pTRAP. All constructs were confirmed by sequencing.

### Transfections and SEAP assays

HeLa, 293 and 293T cells were maintained in Iscove's modified Dulbecco's media (IMDM) supplemented with 10% fetal bovine serum (FBS), 50 μg/ml gentamycin sulfate and 2.5 μg/ml amphotericin B. For transient expression studies, vectors were introduced by calcium phosphate transfection [63]. Transfections using HIV-1 provirus, were performed using 1:2:0.5 ratio (provirus : CMVmyc expression construct : CMVPLAP). Transfections using HIV-1 expression constructs were performed at 1:4:0.4 ratio (HIV-1 expression construct (pgTat and derivatives thereof or GagRRE) : CMVmyc expression construct : Rev expressing construct), depending on the experiment either 0.4 μg VA or 0.5 μg CMVPLAP was also included.

Cell lysates were prepared by harvesting cells 48 hrs post transfection in either RIPA buffer (50 mM Tris-HCl pH 7.5, 1% NP40, 0.05% SDS, 0.5% Sodium Deoxycholate, 1 mM EDTA, 150 mM NaCl) followed by shaking for 20 mins at room temperature and pelleting by centrifugation or 9 M urea, 5 mM Tris pH8 followed by boiling for 10 mins and pelleting by centrifugation. Total RNA was prepared by lysing cells in 4 M GT solution as described in [64]. The RNA was treated for 30 mins at 30°C with TURBO DNase as outlined by the manufacturer (Ambion), then extracted for a second time in 4 M GT solution as before.

Secreted alkaline phosphatase (SEAP) levels were assayed by diluting an aliquot of cell media in water and adding 10 mM diethanolamine (pH 9.5, 0.5 mM MgCl_2_) containing 4-nitrophenyl phosphate (1 mg/ml). SEAP levels were measured at 405 nm using a Titertek multiscan^® ^Plus ELISA plate reader.

### Western blots

Proteins were fractionated on sodium dodecyl sulfate-7% (gp160/120) or 10% polyacrylamide gels (Rev, p24, hnRNP E1, hnRNP E2, myc and tubulin) and transferred to PVDF membrane (PALL Corporation). Blots were probed with antibody to p24 (hybridoma line 183-H12-5C), gp160/120 (kindly provided by H. Schaal, Heinrich-Heine-University of Duesseldorf, Germany), hnRNP E1 (Santa Cruz Biotechnology Inc), hnRNP E2 (Santa Cruz Biotechnology Inc), myc (hybridoma line 9E10), Rev (rabbit polyclonal raised against recombinant rev) and tubulin (Sigma) and detected using HRP-conjugated anti-mouse (p24, gp120, myc and tubulin; Jackson ImmunoResearch Laboratories Inc), anti-rabbit (Rev; Jackson ImmunoResearch Laboratories Inc) or anti-goat (hnRNP E1 and hnRNP E2; Santa Cruz Biotechnology Inc) antibody and the Western Lightning kit (Perkin-Elmer). To quantitate changes in Rev protein expression, developed films were scanned and analyzed using Imagequant software.

### siRNA transfections

293 cells were washed with media minus serum/antibiotics and transfected with 10 nM final concentration of 27mer siRNAs (IDT) against hnRNP E1 (E1 (3): 5'CUU GAA UCG AGU AGG CAU CUA GAG3'; E1 (16): 5' GUA CUG UUG GUC AUG GAG CUG UUG AUA 3'), hnRNP E2 (5' AGA CUG UUG CAU UGC CAA CUG GUG CAG 3') or a scrambled negative control (5' CUU CCU CUC UUU CUC UCC CUU GGA 3'). 10 μM siRNA was combined with OPTI-MEM (Gibco) and transfected with Oligofectamine™ Reagent (Invitrogen) as per manufacturer's instructions. The siRNA/Oligofectamine solutions were added to cell media minus serum and antibiotics to a final volume of 1 ml. 5 hrs later, the transfection media was removed and replaced with growth media. The following day, cells were transfected with HxBruR-/RI- provirus and CMVPLAP at a ratio of 1:0.5 using Fugene (Roche) as described in the manufacturers instructions. Two days post transfection the cells were harvested and lysed in 9 M urea, 5 mM Tris pH8.

### Immunofluorescence

HeLa cells were plated on glass coverslips, transfected, and processed as previously described [65]. Briefly, cells were processed 48 hrs post transfection by washing with 1× PBS and fixing in 4% paraformaldehyde, 1× PBS for 30 min at room temperature. Cells were washed twice with 1× PBS, 10 mM glycine and permeabilized with 1% Triton X-100 in 1× PBS for 5 min. Cells were washed twice with 1× PBS, 10 mM glycine and blocked in 3% BSA overnight at 4°C. Coverslips were inverted onto primary antibody and incubated for 1 hr at room temperature. After washing twice in 1× PBS, 10 mM glycine, coverslips were inverted over fluorescently (FITC and Texas Red; Jackson ImmunoResearch Laboratories Inc) labeled secondary antibody and incubated for 1 hr. Samples were washed with 1× PBS, 10 mM glycine, and stained for DAPI by washing in 1× PBS, DAPI (50 ng/ml) for 10 min. Coverslips were mounted in 90% glycerol and analyzed using a Leica DMR epifluorescent microscope.

### RNA analysis

RNase protection assays (RPAs) were performed as described previously using 10 μg of total RNA [17]. Alternatively, nuclear and cytoplasmic fractions were prepared as previously described [17] prior to RNA extraction. Three RPA probes were used. The rev probe was a BamH1/XhoI fragment of HXB-2 cloned into Bluescript. The env and VA probes are described elsewhere (env probe: [45]; VA probe: [16]). Position of bands was determined following exposure to phosphor screens and scanning using a Phosphor Imager.

For Northern blots, RNA (20 μg) was run on a 1% formaldehyde agarose gel in 1× gel running buffer (0.1 M MOPS pH7, 40 mM NaAc, 5 mM EDTA pH8). The RNA was transferred to nitrocellulose membrane (HYBOND N+, Amersham) overnight in 5 × SSC, 10 mM NaOH and immobilized by UV crosslinking. Membranes were prehybridized at 50°C for 2 hrs in 20 ml Church buffer (0.5 M Phosphate buffer, 7% SDS, 1 mM EDTA) and hybridized with probe overnight at 50°C. Probe to HIV LTR was designed to the LTR region found in all 3 classes of HIV-1 RNA and generated by PCR using the primers (F 5'CTA ATT CAC TCC CAA CGA AGA 3'; R 5' TGC TAG AGA TTT TCC ACA CTG 3'). GAPDH probe was generated by end labeling the primer (5' AAA GGT GGA GGA GTG GGT GTC GCT GTT GAA 3') with ^32^P γATP. Membranes were washed in 6 × SSC for 5 mins, 6 × SSC, 0.1%SDS for 15 mins and 2 × SSC, 0.1%SDS for 15 mins all at 45°C, followed by a brief wash in 2 × SSC at room temperature before exposure to a phosphor screen. Membranes were stripped by washing in boiling 0.1% SDS for 5 mins followed by rinsing in 2 × SSC at room temperature.

For RT-PCR, cDNA was generated by incubation of 3 μg total RNA with random hexamer and M-MLV RT as outlined by the manufacturer (Invitrogen). Separate RT-PCRs were performed for the 2 kb and 4 kb classes of HIV-1 RNA. For the 2 kb class of RNA, 1 μl of cDNA was incubated with 10 μM Forward primer Odp045 (5' CTG AGC CTG GGA GCT CTC TGG C 3') and 10 μM reverse primer Odp032 (5'CCG CAG ATC GTC CCA GAT AAG 3') and Taq. Reactions were heated at 94°C for 2 mins, then cycled at 94°C for 1 min, 57°C for 1 min, 68°C for 1 min (repeated 34 times) and 68°C for 5 mins. A 1/10 dilution of the PCR product was prepared and 3 μl used as the template for a second PCR containing 10 μM forward and reverse primers with Taq as before however this time 0.5 μl α^32^P dCTP was included. The second PCR was cycled at 94°C for 2 mins, then at 94°C for 1.5 mins, 57°C for 1 min, 68°C for 1 min (repeated 3 times) and 68°C for 5 mins.

To analyze the 4 kb class of HIV RNA, 3 μl of cDNA was incubated with the forward primer Odp045 and reverse primer Odp084 (5' TCA TTG CCA CTG TCT TCT GCT CT 3') and Taq and cycled as indicated above. For the second PCR, 3 μl of undiluted RT-PCR reaction was used as the template and cycled with primers Odp045 and Odp084, as described for the 2 kb class of RNAs.

### RNA affinity chromatography and mass spectrometry

pTRAP ESS and pTRAP S5-2 were linearized with XhoI and *in vitro *transcribed with T7 RNA polymerase. Streptavidin beads (62.5 μl) were pre-bound to 5–10 μg of RNA for 30 mins at 4°C. Pre-cleared HeLa nuclear extract (120 μl, 10 mg/ml) containing 0.5 M ATP, 40 mM creatine phosphate, 4 mM MgCl_2 _and 4 mM DTT was subsequently added to the Streptavidin beads and incubated for 1 hr at 4°C. Beads were washed three times in 1× TBP (60 mM HEPES, 10 mM NaCl and 0.1% Triton × pH 7.4). Elutions were performed with 250 μl Biotin elution buffer (5 mM Biotin in 1× TBP) and collected proteins precipitated with acetone.

Column fractions were separated on SDS-PAGE gels and detected by silver staining or Coomassie blue. Bands enriched in the wildtype ESS3a sample were excised, and destained in 50% 100 mM NH_4_HCO_3_/50% acetonitrile. Gel slices were washed with 100 mM NH_4_HCO_3_, 1 mM DTT, then 100 mM NH_4_HCO_3_, 1 mM CaCl_2 _and finally dehydrated in acetonotrile. After drying to remove residual acetonitrile, gel was immersed in 100 mM NH_4_HCO_3_, 12.5 ng/μl trypsin for 45 min. on ice. An equal volume of 100 mM NH_4_HCO_3_, 1 mM CaCl_2 _was subsequently added and digest continued overnight at 30°C. Following digestion, peptides were separated and eluted with an organic gradient using a reverse phase C18 microcapillary column and analyzed in real time with a linear ion trap tandem mass spectrometer (LTQ, ThermoFinnigan) with a custom-built ion source. Precursor peptide ions were automatically selected for data-dependentfragmentation using dynamic exclusion. The resulting tandem mass spectra were searched against the full set of predicted ORFs downloaded from SGD using a distributed version of Sequest. Confidence scores were assigned and high-confidence protein identifications (>95% likelihood) were filtered using the Statquest probability algorithm [66].
